# Recombinant Mouse PAP Has pH-Dependent Ectonucleotidase Activity and Acts through A_1_-Adenosine Receptors to Mediate Antinociception

**DOI:** 10.1371/journal.pone.0004248

**Published:** 2009-01-22

**Authors:** Nathaniel A. Sowa, Kunjumon I. Vadakkan, Mark J. Zylka

**Affiliations:** Department of Cell and Molecular Physiology, UNC Neuroscience Center, University of North Carolina, Chapel Hill, North Carolina, United States of America; Emory University, United States of America

## Abstract

Prostatic acid phosphatase (PAP) is expressed in nociceptive neurons and functions as an ectonucleotidase. When injected intraspinally, the secretory isoforms of human and bovine PAP protein have potent and long-lasting antinociceptive effects that are dependent on A_1_-adenosine receptor (A_1_R) activation. In this study, we purified the secretory isoform of mouse (m)PAP using the baculovirus expression system to determine if recombinant mPAP also had antinociceptive properties. We found that mPAP dephosphorylated AMP, and to a much lesser extent, ADP at neutral pH (pH 7.0). In contrast, mPAP dephosphorylated all purine nucleotides (AMP, ADP, ATP) at an acidic pH (pH 5.6). The transmembrane isoform of mPAP had similar pH-dependent ectonucleotidase activity. A single intraspinal injection of mPAP protein had long-lasting (three day) antinociceptive properties, including antihyperalgesic and antiallodynic effects in the Complete Freund's Adjuvant (CFA) inflammatory pain model. These antinociceptive effects were transiently blocked by the A_1_R antagonist 8-cyclopentyl-1, 3-dipropylxanthine (CPX), suggesting mPAP dephosphorylates nucleotides to adenosine to mediate antinociception just like human and bovine PAP. Our studies indicate that PAP has species-conserved antinociceptive effects and has pH-dependent ectonucleotidase activity. The ability to metabolize nucleotides in a pH-dependent manner could be relevant to conditions like inflammation where tissue acidosis and nucleotide release occur. Lastly, our studies demonstrate that recombinant PAP protein can be used to treat chronic pain in animal models.

## Introduction

Small-diameter dorsal root ganglia (DRG) neurons contain a classic, histochemically-defined enzyme known as Fluoride-Resistant Acid Phosphatase (FRAP) or Thiamine Monophosphatase (TMPase) [1], [2]. Recently, we found that TMPase was molecularly equivalent to Prostatic Acid Phosphatase (PAP, also known as ACPP) [3]. In mammals, PAP is expressed as a secreted protein or as a transmembrane protein [4], [5], [6]. These isoforms have identical N-terminal regions, including a signal peptide and extracellular acid phosphatase domain, but differ at the C-terminus due to the inclusion or exclusion of a transmembrane domain. Using in situ hybridization with isoform-specific riboprobes, we found that small-diameter DRG neurons primarily express the transmembrane isoform of PAP [3]. Moreover, using immunohistochemistry, we found that PAP protein is localized to a majority of all nonpeptidergic nociceptive neurons, a subset of peptidergic nociceptive neurons and to axon terminals located in lamina II of the dorsal spinal cord [3].

We also found that PAP functions in nociceptive circuits as an ectonucleotidase by dephosphorylating adenosine monophosphate (AMP) to adenosine [3]. This was based on our observation that intrathecal injection of human (h)PAP protein (the secreted isoform) produced long-lasting antinociceptive, antihyperalgesic and antiallodynic effects that were dependent on A_1_-adenosine receptor (A_1_R) activation [3]. These antinociceptive effects were eight-times more effective than the commonly used analgesic morphine. When injected intrathecally, bovine (b)PAP also had long-lasting antinociceptive effects that were dependent on A_1_R activation. Conversely, PAP knockout (*PAP^−/−^*) mice showed enhanced sensitivity in animal models of chronic inflammatory pain and neuropathic pain [3], a phenotype that was similar to *A_1_R^−/−^* mice [7]. Lastly, dephosphorylation of extracellular AMP was greatly reduced in small-diameter DRG neurons and dorsal spinal cord of *PAP^−/−^* mice.

For our initial study, we used secretory isoforms of PAP that were purified from human seminal fluid and from bovine prostate [3]. The secretory isoforms of human, bovine and mouse PAP are ∼80% identical to one another at the amino acid level (based on pairwise sequence comparisons), suggesting they might have similar antinociceptive effects in vivo. At the time we performed our initial studies, we were unable to test mPAP protein for antinociceptive effects because there were no commercially available sources of pure mPAP protein. Moreover, without pure protein, we could not determine the substrate specificity for secretory mPAP. To overcome these limitations, we synthesized and purified recombinant mPAP protein (secretory isoform). Strategies for generating recombinant human and rat PAP protein were previously described [8], [9]. At neutral pH, mPAP primarily dephosphorylated AMP. In addition, we found that mPAP could dephosphorylate all purine nucleotides (AMP, ADP, ATP) under acidic pH conditions. This suggested a broader function for PAP in nucleotide metabolism and has implications in inflammatory pain conditions where extracellular pH is reduced.

Recombinant proteins can be produced in large quantities, are not likely to be contaminated with human pathogens and can be used in humans [10], [11], [12]. Thus, the approaches outlined in this study could be used to purify and test recombinant mouse or human PAP as a treatment for chronic pain in humans.

## Results

### Purification of recombinant mPAP using the baculovirus expression system

**Figure 1:**
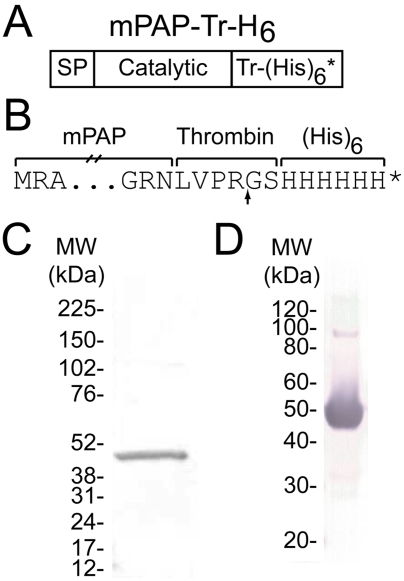
Purification of recombinant mPAP. (A) A thrombin cleavage site (Tr) followed by hexahistidine tag (H_6_) and stop codon (*) were added to the C-terminus of the secretory isoform of mPAP. SP = signal peptide of mPAP. Map is not drawn to scale. (B) Amino acid sequence at the junction between the catalytic domain and Tr-H_6_ tag. Arrow marks thrombin cleavage site. Asterisk marks stop codon. (C) GelCode blue-stained SDS-PAGE gel and (D) western blot of purified recombinant mPAP protein (1 µg and 5 µg, respectively). The western blot was probed with an anti-hexahistidine antibody.

Large quantities of recombinant human or rat PAP (secretory isoform) can be generated in yeast or baculovirus expression systems [8], [9]. We generated a baculovirus expression construct containing the entire open-reading frame of secretory mPAP, encompassing the signal peptide (SP) and catalytic domain fused to a C-terminal thrombin-hexahistidine (Tr-H_6_) epitope tag (Fig. 1A, B). Although the thrombin cleavage site can be used to efficiently remove the epitope tag (Fig. 1B, data not shown), we performed our studies below with recombinant mPAP-Tr-H_6_ (henceforth referred to as mPAP) containing the C-terminal epitope tag because removal of the tag required additional purification steps and did not impact enzyme activity.

**Figure 2:**
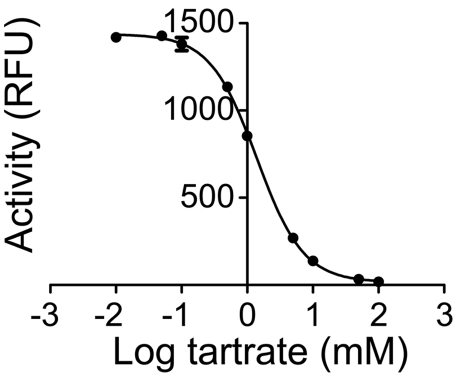
Inhibition of mPAP by L-(+)-tartrate. The indicated concentrations of L-(+)-tartrate were added to reactions (n = 3 per concentration) containing mPAP (1 U/mL), 100 mM sodium acetate, pH 5.6 and the fluorescent acid phosphatase substrate DiFMUP. Relative fluorescence units (RFU). All data are presented as means±s.e.m. Prism 5.0 (GraphPad Software, Inc) was used to generate curve.

We detected large quantities of mPAP protein in the tissue culture supernatant of Hi5 insect cells two days after infection with recombinant baculovirus. We purified mPAP from the supernatant in one step, using nickel chelate affinity chromatography. We confirmed protein purity by running mPAP on an SDS-PAGE gel and staining for total protein (Fig. 1C) and western blotting (Fig. 1D). In both cases, we observed one predominant band at ∼45 kDa, corresponding to the calculated molecular weight of monomeric mPAP (45.2 kDa). The weakly stained ∼90 kDa band on our overloaded western blot likely reflects a small amount of non-denatured mPAP, consistent with the fact that native PAP is a dimer [13], [14]. No additional bands were observed, indicating that mPAP protein was pure and largely intact. This purified, recombinant mPAP protein effectively dephosphorylated the generic acid phosphatase substrates para-nitrophenyl phosphate (p-NPP) and 6,8-difluoro-4-methylumbelliferyl phosphate (DiFMUP) and was inhibited by the acid phosphatase inhibitor L-(+)-tartrate (IC_50_ = 1.45 mM; Fig. 2). Recombinant human and rat PAP are similarly inhibited by L-(+)-tartrate [8], [9], [15].

### Recombinant mPAP dephosphorylates purine nucleotides in a pH-dependent manner

**Figure 3:**
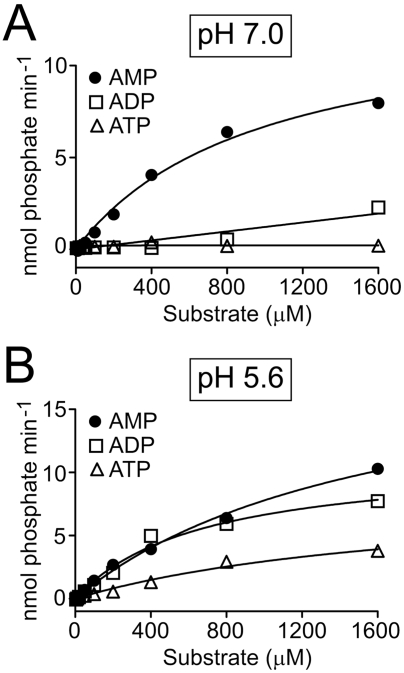
mPAP dephosphorylates purine nucleotides in a pH-dependent manner. Plot of initial velocity at the indicated concentrations of AMP, ADP and ATP at (A) pH 7.0 and (B) pH 5.6. Reactions (n = 3 per point) were stopped after 3 min. Inorganic phosphate was measured using malachite green. All data are presented as means±s.e.m. Error bars are obscured due to their small size.

We previously found that hPAP (secreted isoform) generated adenosine by dephosphorylating AMP and, to a much lesser extent, ADP at neutral pH [3]. To determine if secretory mPAP had similar substrate specificity and to evaluate pH dependence, we incubated mPAP with AMP, ADP or ATP at pH 7.0 or pH 5.6, then detected inorganic phosphate using the malachite green assay. We found that mPAP dephosphorylated AMP and, to a lesser extent, ADP at neutral pH (Fig. 3A), consistent with our previous findings using hPAP [3]. At pH 5.6, mPAP dephosphorylated AMP and ADP, and to a lesser extent, ATP (Fig. 3B). This latter finding was consistent with a previous study showing that secretory hPAP could dephosphorylate all nucleotides under acidic conditions with a rank order AMP>ADP>ATP [16].

**Figure 4:**
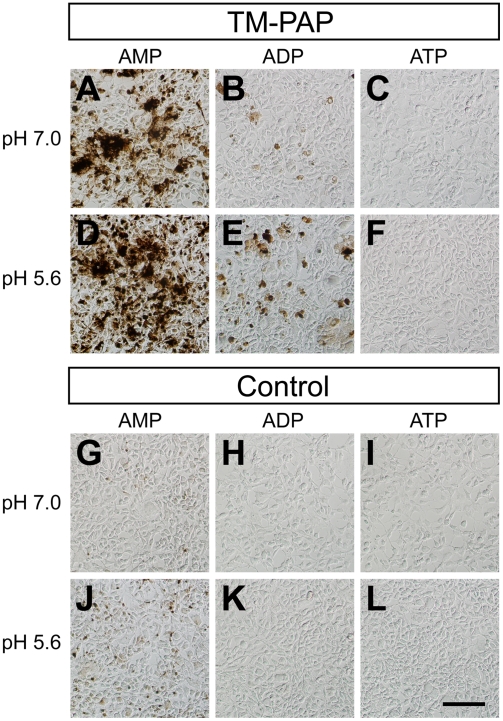
TM-PAP dephosphorylates extracellular purine nucleotides in a pH-dependent manner. HEK 293 cells were transfected with expression vectors containing (A–F) mouse TM-PAP or (G–L) the fluorescent protein Venus as a control. Cells were then histochemically stained using AMP, ADP or ATP (each 6 mM) as substrate at pH 7.0 or pH 5.6. Cells were not permeabilized with detergent. Scale bar (bottom right panel), 50 µm for all panels.

We previously found that the transmembrane isoform of mouse PAP (TM-PAP) could dephosphorylate extracellular AMP at pH 5.6 using enzyme histochemistry, indicating that PAP had ectonucleotidase activity [3]. At the time, we did not test hydrolysis at neutral pH or hydrolysis of other nucleotides. To determine if TM-PAP could dephosphorylate additional nucleotides extracellularly, and if dephosphorylation was pH dependent, we transfected mouse TM-PAP into HEK 293 cells and stained non-permeabilized cells using enzyme histochemistry. Use of non-permeabilized cells allowed us to measure extracellular nucleotide hydrolysis in a cellular context. At pH 7.0, TM-PAP transfected cells were heavily stained using AMP as substrate and much less intensely stained using ADP as substrate (Fig. 4A–C). At pH 5.6, TM-PAP transfected cells were heavily stained using AMP and moderately stained using ADP as substrate (Fig. 4D–F). Control cells transfected with the fluorescent protein Venus were not intensely stained under any of the conditions examined (Fig. 4G–L). When taken together, these data suggest TM-PAP has pH-dependent ectonucleotidase activity, with AMP being the preferred substrate at neutral pH and AMP and ADP being substrates at acidic pH. Moreover, these data suggest PAP could generate adenosine following hydrolysis of AMP, ADP or ATP under acidic pH conditions [3].

### Recombinant mPAP has long-lasting antinociceptive properties

**Figure 5:**
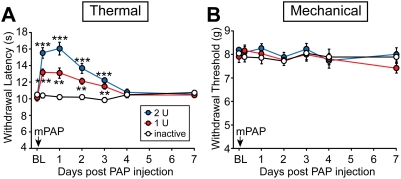
Dose-dependent antinociceptive effects of intrathecal mPAP. (A) Effects of increasing amounts of mPAP on paw withdrawal latency to a radiant heat source. (B) Paw withdrawal threshold to a semi-flexible tip mounted on an electronic von Frey apparatus. (A, B) BL = Baseline. Injection (i.t.) volume was 5 µL. n = 8 wild-type mice were used per dose. There were significant differences over time between mice injected with heat-inactivated (0 U) mPAP and mice injected with active (1 U or 2 U) mPAP (Repeated measure two-way ANOVA; *P*<0.0001 for each dose). Post-hoc paired t-tests were used to compare responses at each time point between mice injected with active mPAP to mice injected with heat-inactivated mPAP (** *P*<0.005; *** *P*<0.0005). For the heat-inactivated mPAP control, the protein concentration was equivalent to the maximum 2 U dose of mPAP (1.1 mg/mL). All data are presented as means±s.e.m.

A single intrathecal injection of hPAP protein (secreted isoform) has antinociceptive, antihyperalgesic and antiallodynic effects that last for three days [3]. To determine if mPAP also had long-lasting antinociceptive effects, we intrathecally (i.t.) injected wild-type mice with two doses of recombinant mPAP protein (Fig. 5). Control mice were injected i.t. with heat-denatured, and hence phosphatase-inactive, mPAP. We then measured noxious thermal and mechanical sensitivity before (baseline, BL) and after mPAP injections. Six hours after i.t. injection, paw withdrawal latency to the noxious thermal stimulus was significantly increased relative to controls and remained elevated for three days (Fig. 5A). This antinociceptive effect was dose-dependent and required catalytic activity, as evidenced by loss of antinociception upon heat-inactivation of mPAP (Fig. 5A). Active mPAP did not alter mechanical sensitivity (Fig. 5B) nor did it cause paralysis or sedation.

**Figure 6:**
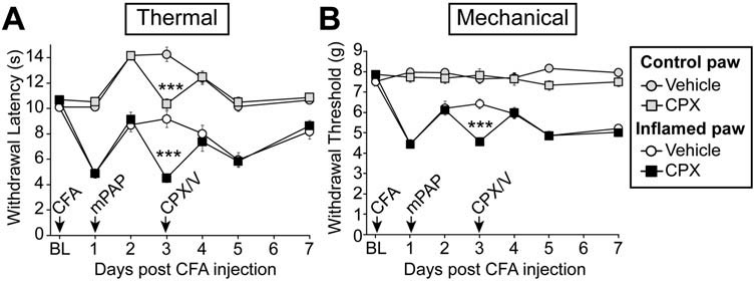
The antinociceptive effects of mPAP can be transiently inhibited with a selective A_1_R antagonist. Wild-type mice were tested for (A) noxious thermal and (B) mechanical sensitivity before (baseline, BL) and following injection of CFA (CFA-arrow) into one hindpaw. The non-inflamed hindpaw served as control. All mice were injected with active mPAP (mPAP-arrow; 2 U, i.t.). Two days later, half the mice were injected with vehicle (CPX/V-arrow, circles; intraperitoneal (i.p.); 1 hr before behavioral measurements) while the other half were injected with 8-cyclopentyl-1, 3-dipropylxanthine (CPX/V-arrow, squares; 1 mg/kg i.p.; 1 hr before behavioral measurements). There were significant differences over time between mice injected with vehicle and mice injected with CPX (Repeated measure two-way ANOVA; *P*<0.01). Post-hoc paired t-tests were used to compare responses at each time point between vehicle (n = 10) and CPX-injected mice (n = 10); same paw comparisons. *** *P*<0.0005. All data are presented as means±s.e.m.

We next tested mPAP for antihyperalgesic and antiallodynic effects using the CFA inflammatory pain model. To do this, we injected CFA into one hindpaw to induce inflammation and used the non-inflamed paw as a control. Intrathecal injection of mPAP produced a significant increase in withdrawal latency to the noxious thermal stimulus (relative to latency on Day 1, pre-injection) in the inflamed paw (Fig. 6A, white open circles). This antihyperalgesic effect persisted for three days. mPAP also caused a significant increase in paw withdrawal latency in the non-inflamed paw (Fig. 6A, grey open circles, relative to day 1 values), reproducing results presented in Fig. 5A. In addition, mPAP produced a significant increase in withdrawal threshold to the mechanical stimulus (relative to latency on Day 1, pre-injection) only in the inflamed paw (Fig. 6B, white open circles). This antiallodynic effect lasted for three days.

### mPAP acts through A_1_Rs to mediate antinociception

The antinociceptive, antihyperalgesic and antiallodynic effects of hPAP are dependent on A_1_R activation [3]. Since our biochemical experiments suggested that mPAP could generate adenosine by dephosphorylating nucleotides, we next evaluated whether mPAP had antinociceptive properties that were dependent on A_1_R activation. To do this, we injected a second group of CFA-inflamed mice with the selective A_1_R antagonist 8-cyclopentyl-1, 3-dipropylxanthine (CPX; 1 mg/kg i.p.). CPX transiently antagonized all antinociceptive effects of mPAP, including the antihyperalgesic (Fig. 6A) and antiallodynic (Fig. 6B) effects. This same i.p. dose of CPX did not affect thermal or mechanical sensitivity in the control or CFA-inflamed paw once the antinociceptive effects of PAP wore off (see Figure S10 in [3]). Taken together, these data suggest that the antinociceptive effects of mPAP were due to ectonucleotidase-dependent generation of adenosine followed by activation of A_1_Rs.

## Discussion

We previously found that PAP was expressed in nociceptive neurons and functioned as an ectonucleotidase by dephosphorylating AMP to adenosine. Moreover, PAP had antinociceptive properties that were dependent on A_1_R activation [3]. At the time, we could not perform in vivo gain-of-function studies with mPAP because there were no commercially available sources of secretory mPAP protein. To overcome this limitation, we generated and purified recombinant mPAP protein and then studied the biochemical properties of mPAP and the effects of mPAP on pain sensitivity.

Our studies revealed that recombinant mPAP has very similar biochemical properties when compared to PAP from other mammalian species, including human [8], [13]. Both mPAP and hPAP are inhibited by L-(+)-tartrate (Fig. 2), both predominantly dephosphorylate AMP at neutral pH (Fig 3A; [3]) and both dephosphorylate all adenine nucleotides (with relative activity AMP>ADP>ATP) at acidic pH (Fig. 3B) [16]. The Km values (0.9–1.6 mM) we obtained for mPAP using AMP as substrate were within the range of Km values (0.37–2 mM) reported for hPAP using AMP as substrate [17], [18], [19]. Mouse TM-PAP also dephosphorylated extracellular adenine nucleotides in a pH-dependent manner, although ATP was not a substrate for TM-PAP as it was for secretory PAP. This substrate discrepancy could reflect biochemical differences between these isoforms. Or, more likely, this reflects reduced sensitivity of the histochemical assay relative to the in vitro enzyme assay. When taken together, our findings suggest PAP functions as an ecto-5′-nucleotidase (with relative selectivity for AMP) at neutral pH and as a generic ectonucleotidase (with selectivity for AMP, ADP and ATP) at acidic pH.

This pH-dependent hydrolysis of purine nucleotides is intriguing, especially when considering that tissue injury produces an “inflammatory soup” containing protons and nucleotides [20]. Protons produce tissue acidosis, modulate the capsaicin receptor TRPV1 and activate acid-sensing ion channels (ASICs) half-maximally at pH values ranging from 4.9 to 6.8 [21], [22], [23]. ATP and ADP activate purinergic P2X and P2Y receptors [24], [25]. Stimulation of these diverse receptors sensitizes nociceptive neurons, activates spinal microglia and causes pain [24], [26], [27], [28], [29]. PAP is extensively co-localized with the ATP receptor P2X3 and is co-localized in 14.4% of all TRPV1^+^ DRG neurons in the mouse [3]. Since PAP protein is localized on peripheral terminals of these neurons [3] and can dephosphorylate adenine nucleotides at acidic pH, PAP could metabolize pain-producing ATP and ADP in the inflammatory soup and reduce the subsequent sensitization of nociceptive neurons. This is consistent with our observation that *PAP^−/−^* mice show enhanced thermal hyperalgesia and mechanical allodynia following inflammation [3].

In addition, PAP is localized on the central terminals of nociceptive neurons [3] and could metabolize nucleotides to adenosine in a pH-dependent manner at central synapses. The pH of synaptic vesicles is 5.6±0.7 [30] and intense neural activity can lead to acidosis within synapses that lasts for seconds [23]. Likewise, inflammation, tissue injury and repetitive stimulation cause acidosis of up to 0.25 pH units in the dorsal horn of spinal cord when measured with pH-sensitive microelectrodes [31], [32], [33]. Considering the size of these microelectrodes relative to the small volume of a synapse, these microelectrode recordings likely underestimate the magnitude of the pH change that occurs within the confines of a synapse. Thus, PAP may be exposed to low extracellular or endosomal pH when spinal synapses are activated for sustained periods of time.

Intrathecal injection of mPAP produced dose-dependent, potent and long-lasting (3 days) antihyperalgesic effects that were specific for the thermal modality in uninjured animals (Fig. 5) and antihyperalgesic and antiallodynic effects in CFA-inflamed animals (Fig. 6). Likewise, hPAP and bPAP had similar antinociceptive effects lasting three days and two days, respectively [3]. And, just like hPAP and bPAP mediated antinociception, A_1_R receptor activation was required for mPAP mediated antinociception. When combined with our biochemical results, this suggests that mPAP converts extracellular nucleotides to adenosine in vivo. Moreover, these data suggest a species-conserved function for human, bovine and mouse PAP as an ectonucleotidase.

Adenosine and A_1_R agonists have potent and, in some studies, long-lasting (>24 h) analgesic effects in rodents and humans when injected peripherally or centrally [26], [34], [35]. However, adenosine and A_1_R agonists are not used clinically to treat chronic pain because of side-effects, including transient lower back pain [36], [37], and motor paralysis when administered at high doses [26]. Motor side-effects could be due to widespread expression of A_1_R throughout the spinal cord, including relatively high-level expression in motor neurons [38].

We did not observe motor paralysis at the highest doses of mouse and human PAP tested, despite the fact that PAP also works via A_1_R activation (Figs. 5, 6) [3]. This could be due to the fact that, as an enzyme, the amount of adenosine produced by PAP is limited by substrate concentration. Thus, through catalytic restriction, PAP may produce sufficient amounts of adenosine to mediate antinociception but not enough adenosine to cause overt motor side-effects.

The resting CSF concentration of AMP in humans is 1.8 µM [39], well below the Km of mPAP and hPAP for AMP. Since this AMP concentration is below Km, PAP could produce linear increases in adenosine as the extracellular AMP concentration increases. This would allow PAP to dynamically generate adenosine over a wide range of nucleotide concentrations. This could be relevant in chronic pain states where extracellular nucleotides are likely to be elevated [40], [41].

Recombinant proteins, such as human growth hormone and interferons, are routinely used to treat a variety of human diseases and disorders [11], [12]. We found that recombinant mPAP protein functions as a pH-dependent ectonucleotidase and has antinociceptive effects in an animal model of inflammatory pain. Unlike direct injections of adenosine and A_1_R agonists which produce antinociception and motor side effects, mPAP injections *indirectly* elevate adenosine levels and produce antinociception without side-effects. Interestingly, other methods that indirectly elevate adenosine, such as using adenosine kinase inhibitors, also produce antinociception without motor side effects [42], [43], [44], [45]. Considering how readily recombinant mPAP and hPAP can be purified [9], and the fact that recombinant hPAP (fused to GM-CSF; also known as PA2024, a component of the Provenge/Sipuleucel-T immunotherapy) is safe to use in humans [10], [46], recombinant PAP could be developed as a protein-based therapeutic for chronic pain. Moreover, it might be possible to further optimize PAP stability and kinetic parameters for therapeutic purposes, using site-directed mutagenesis and the PAP three-dimensional structure as a guide [9], [14], [15], [47].

## Materials and Methods

### Molecular biology and protein purification

The mPAP-Tr-(His)_6_ baculovirus expression clone (encompassing nt 61–1206 from GenBank accession # NM_019807) was generated by PCR amplification, using a full-length expression construct of mPAP (secreted isoform) as template and Phusion polymerase. PCR products were cloned into pFastBac1 (Invitrogen) and completely sequenced. Primer sequences contained XbaI sites (underlined) to facilitate cloning (N-terminal primer: 5′-cgctctagaaccatgcgagccgttcctctgc. C-terminal thrombin-(His)_6_ tag primer: 5′-gcgtctagattaatgatgatgatgatgatgggagccacgcggaaccagattccgtccttggtggctgc). There are no thrombin cleavage sites in the mPAP protein except for the cleavage site we introduced. This vector was then used to generate recombinant mPAP protein using the Bac-to-Bac Baculovirus Expression System (Invitrogen). Briefly, we infected Hi5 insect cells with high-titer recombinant baculovirus, incubated the cells for 48 hours at 27°C, then harvested and concentrated the supernatant containing secreted mPAP protein. Then, mPAP protein was purified from the concentrated supernatant using Ni-NTA HisTrap agarose (GE Healthcare Life Sciences) affinity chromatography and imidazole as eluant. Lastly, mPAP protein was dialyzed against PBS to remove imidazole. Protein purity was confirmed by SDS-PAGE, staining for total protein with GelCode Blue (Pierce/Thermo Scientific, Cat. # 24590) and western blotting with Penta-His antibody (Qiagen, Cat. # 34660). Amersham full-range rainbow molecular weight markers (GE Healthcare) were used for SDS-PAGE and MagicMark XP markers (Invitrogen, Cat. # LC5602) were used for western blots. This purification strategy is based on the observation that recombinant rat PAP is secreted into the medium of baculovirus-infected insect cells [8]. Recombinant mPAP was kept at 4°C for short-term (1–2 months) use and at −80°C for long-term storage.

### Enzyme assays

Enzymatic reactions (50 µL final) were carried out with recombinant mPAP at 37°C for 3 minutes in 10 mM sodium acetate, pH 5.6 or 10 mM HEPES, pH 7.0 with AMP, ADP or ATP as substrate. Reactions were stopped by adding 950 µL of the malachite green color reagent [0.03% (w/v) malachite green oxalate, 0.2% (w/v) sodium molybdate, 0.05% (v/v) Triton X-100, dissolved in 0.7 M HCl] then incubating at room temperature for 30 minutes. Inorganic phosphate was quantified by measuring OD_650_ and comparing to an inorganic phosphate (KH_2_PO_4_) standard curve [48].

Enzyme activity of mPAP was determined using 4-nitrophenyl phosphate as substrate following Sigma's Quality Control Test Procedure for PAP (SSPNPP11, revision 8/29/97). Unit (U) definition: 1 U hydrolyzes 1 µmole of 4-nitrophenyl phosphate per minute at 37°C at pH 4.8.

### Cell culture and histochemistry

HEK 293 cells were cultured and transfected as previously described [3]. Enzyme histochemistry was performed as previously described [3] using 6 mM AMP, ADP, or ATP as substrate and Tris-maleate buffer at pH 5.6 or 7.0.

### Behavior

All behavioral experiments involving vertebrate animals were approved by the Institutional Animal Care and Use Committee at the University of North Carolina at Chapel Hill.

C57BL/6 male mice, 2–4 months old, were purchased from Jackson Laboratories and used for all behavioral experiments. All mice were acclimated to the testing room, equipment and experimenter for at least three days before behavioral testing. The experimenter was blind to drug treatment during behavioral testing.

Thermal sensitivity was measured by heating one hindpaw with a Plantar Test apparatus (IITC) following the Hargreaves method [49]. The radiant heat source intensity (Plantar test apparatus, IITC) was calibrated so that a paw withdrawal reflex was evoked in ∼10 s., on average, in wild-type C57BL/6 mice. Cutoff time was 20 s. One measurement was taken from each paw per time point to determine paw withdrawal latency. Mechanical sensitivity was measured using semi-flexible tips attached to an Electronic von Frey apparatus (IITC) as described elsewhere [50], [51]. Three measurements were taken from each paw (separated at 10 min intervals) then averaged to determine paw withdrawal threshold in grams. To induce inflammatory pain, 20 µL Complete Freunds Adjuvant (CFA, from MP Biomedicals) was injected into one hindpaw, centrally beneath glabrous skin, with a 30G needle. 8-cyclopentyl-1, 3-dipropylxanthine (C101, Sigma) was dissolved in 0.9% saline containing 5% DMSO, 1.25% 1 M NaOH for i.p. injection. Sedation and motor dysfunction were assessed by visually observing motor activity following injections. None of the mPAP-injected mice displayed reduced mobility or paralysis following injection.

### Intrathecal injections

We used concentrated mPAP protein (in PBS; 1.1 mg/mL; 400 U/mL) or diluted mPAP (in 0.9% saline) for injections. mPAP was heat-inactivated by incubating at 65°C for 40 min. Loss of activity was confirmed using the EnzChek Phosphatase Assay Kit (Invitrogen, E12020) following the manufacturer's protocol. Active or heat-inactivated mPAP was intrathecally injected (5 µL) into unanesthetized mice using the direct lumbar puncture method [52].
